# Supplementary material for: Single-cell Micro-C profiles 3D genome structures at high resolution and characterizes multi-enhancer hubs
Source: Nat Genet. 2025 Jul 2;57(7):1777–86. doi: 10.1038/s41588-025-02247-6 (PMC12283367; doi:10.1038/s41588-025-02247-6)
Supplement: Supplementary file 1 — Supplementary Figs. 1–7 and Supplementary Note (protocols). [file 41588_2025_2247_MOESM1_ESM.pdf]

nature genetics

Supplementary information https://doi.org/10.1038/s41588-025-02247-6

Single-cell Micro-C profiles 3D genome structures at high resolution and characterizes multi-enhancer hubs

In the format provided by the authors and unedited

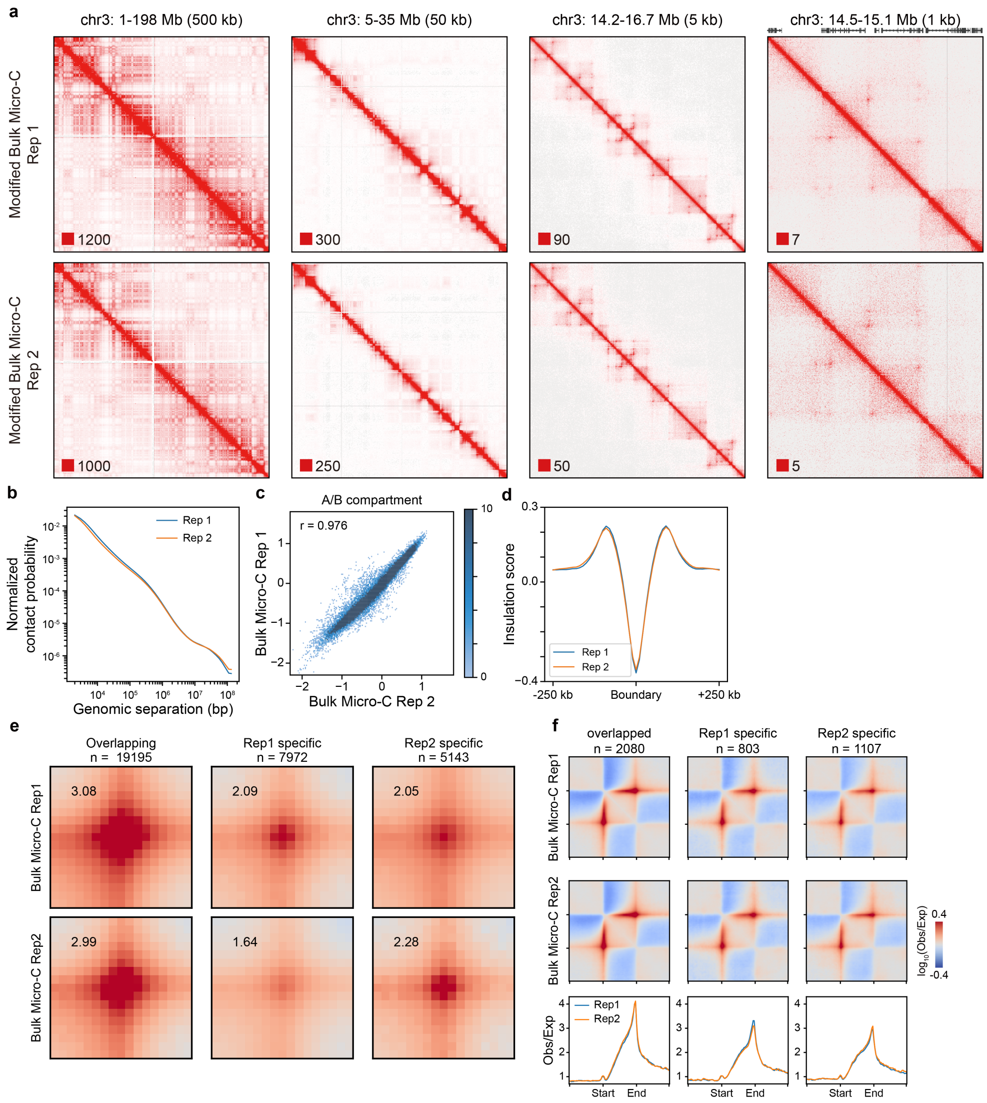

Supplementary Fig. 1. Modified bulk Micro-C validation. a, Contact maps of two bulk Micro-C replicates at different resolutions for GM12878. **b**, Contact frequency as a function of genomic distance for two Micro-C replicates, bulk Micro-C and Hi-C. **c**, Density plot showing the correlation of first eigen values after eigendecomposition at 100 kb resolution between two Micro-C replicates. **d**, Global average insulation scores of TADs boundary for two Micro-C replicates. **e**, Aggregate peak analysis (APA) of chromatin loops for two Micro-C replicates around shared and unique loop sets. **f**, Aggregate plots of chromatin stripes for two Micro-C replicates around shared and unique stripe sets. Chromatin stripes were detected with Stripenn.

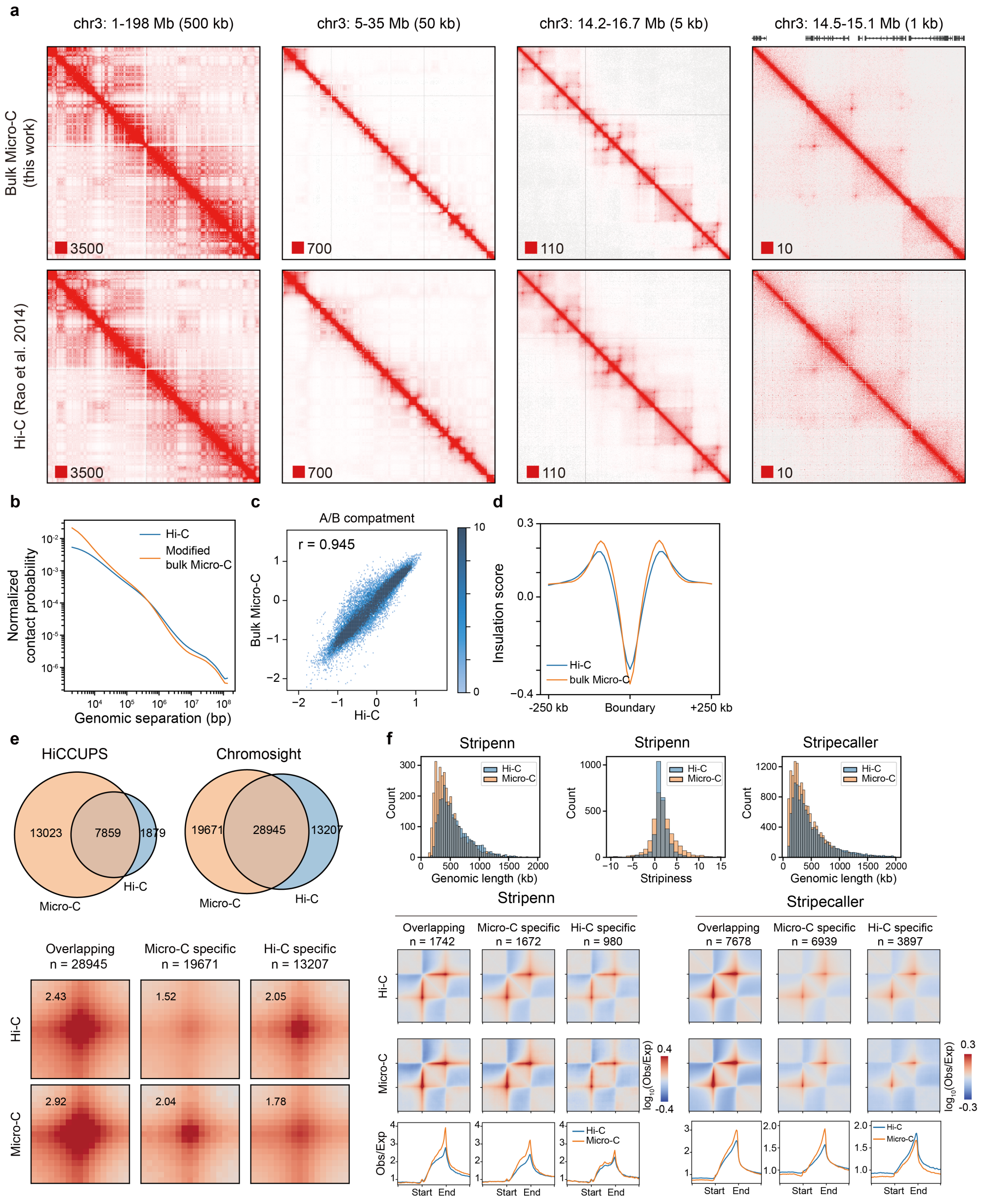

Supplementary Fig. 2. Bulk Micro-C comparison with bulk Hi-C. a, Contact maps of bulk Micro-C and *in situ* Hi-C at different resolutions for GM12878. **b**, Contact frequency as a function of genomic distance for bulk Micro-C and Hi-C. **c**, Density plot showing the correlation of first eigen values after eigendecomposition at 100 kb resolution between bulk Micro-C and Hi-C. **d**, Global average insulation scores of TADs boundary for bulk Micro-C and Hi-C. **e**, Bulk Micro-C show high sensitivity in chromatin loop detection. Top: venn diagram of chromatin loops detected between bulk Micro-C and Hi-C, two loop caller were used, HiCCUPS and Chromsight. Bottom: APA of chromatin loops for bulk Micro-C and Hi-C around shared and specific loop sets. Chromosight detected loop sets were used. **f**, Comparison of detected chromatin stripes between bulk Micro-C and bulk Hi-C, two algorithms were used, Stripenn and Stripecaller.

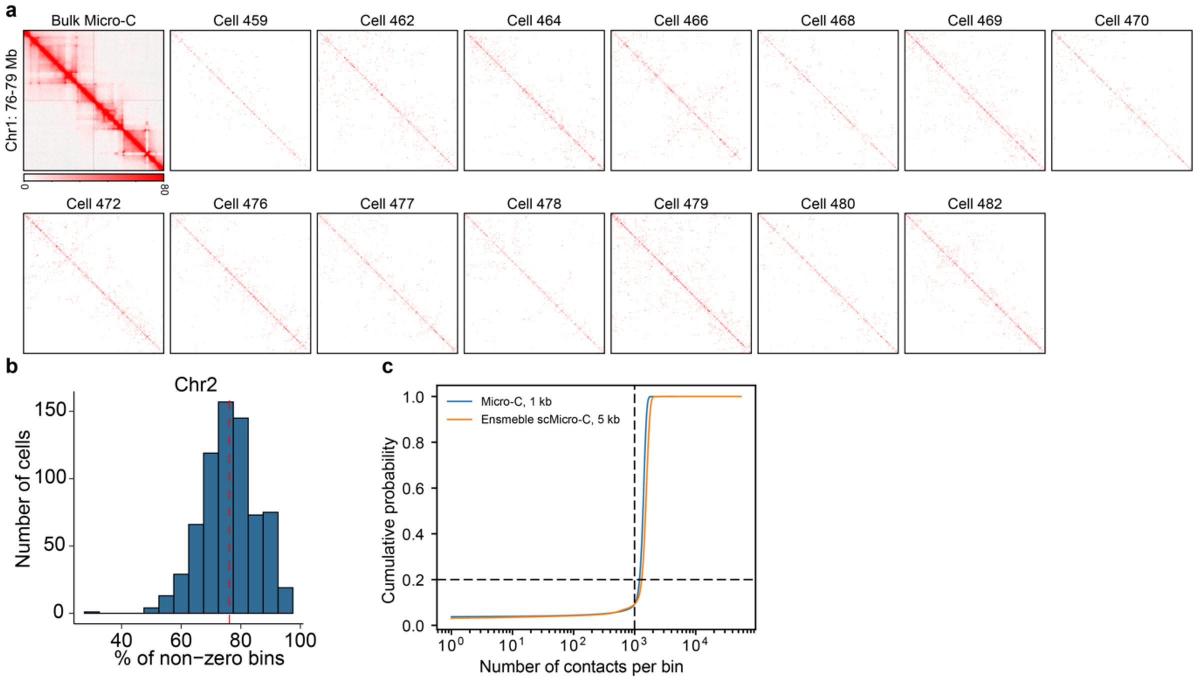

Supplementary Fig. 3. scMicro-C and bulk Micro-C contact map-based resolution. a, bulk Micro-C and representative scMicro-C contact map at 5 kb resolution. **b**, Histogram showing the distribution of non-zero bins of scMicro-C contact map of chromosome 2 at 5 kb resolution. **c**, Cumulative curve of 80% of bins with contacts greater than 1202.

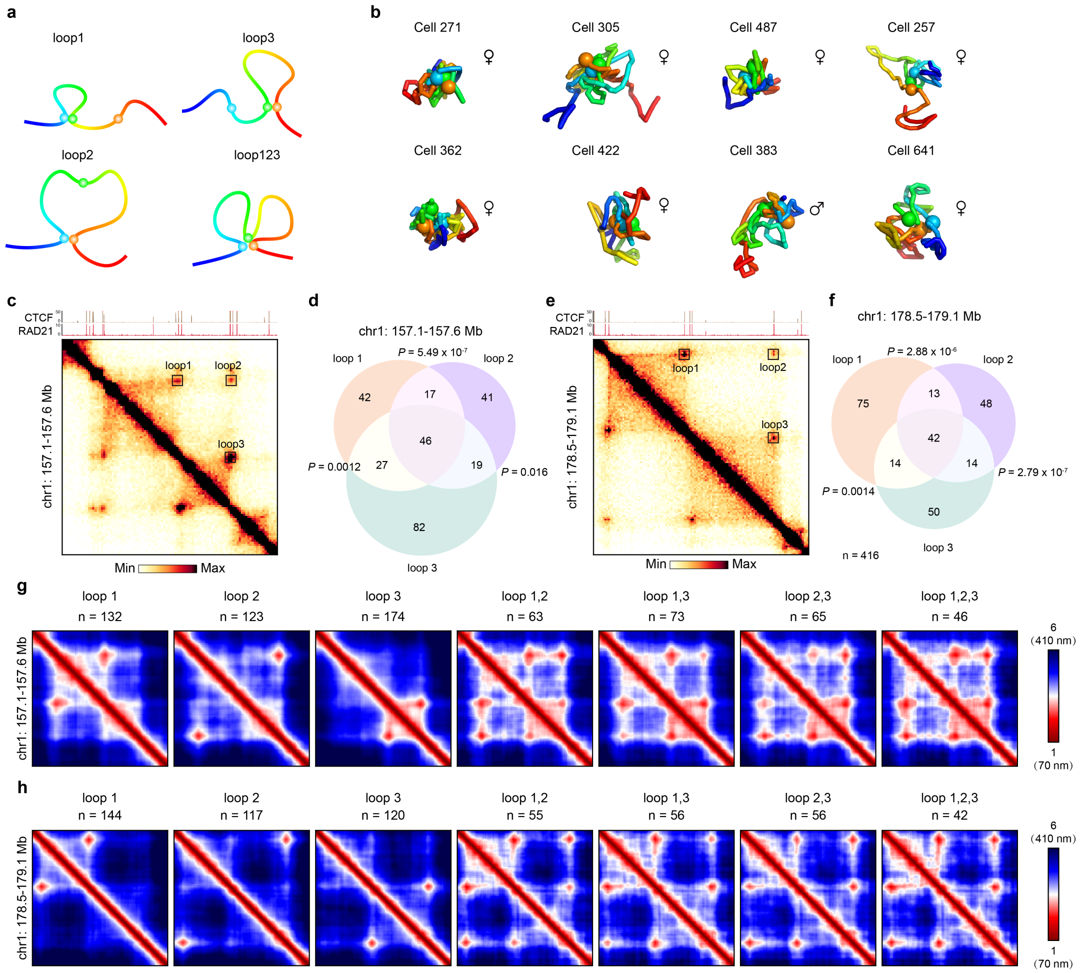

Supplementary Fig. 4. Nested chromatin loops show coordinated interactions. **a**, Schematics showing the loop formation events. **b**, Representative single-cell 3D structures forming nested three-way chromatin loops at chr1: 224.4-225 Mb locus. **c**, Contact map of a nested chromatin loops. **d**, Venn diagram shows the overlap of single-cell 3D structures forming three loops. Hypergeometric test (one-sided) was used, the number of total available structures for this region is 399. **e**, Contact map of another nested chromatin loops. **f**, Venn diagram summarizes the overlap of single-cell 3D structures forming three loops. Hypergeometric test (one-sided) was used, the number of total available structures for this region is 416. **g**, Mean 3D distance matrices of single-cell 3D genome structures forming the corresponding loops (chr1: 157.1-157.6 Mb). **h**, The same as e, for chr1: 178.5-179.1 Mb.

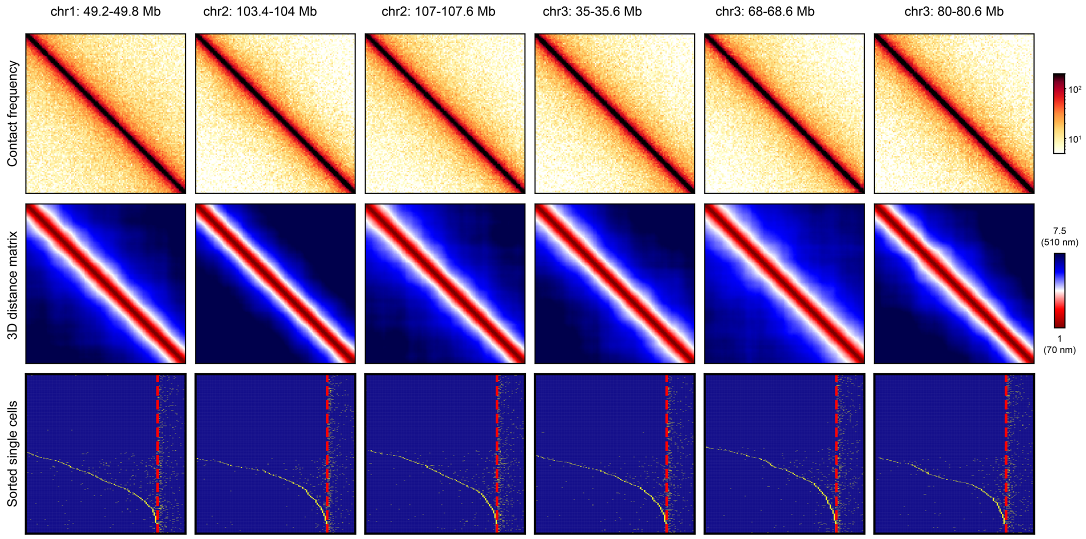

Supplementary Fig. 5. Non-stripe control regions. Top: bulk Micro-C contact maps of six selected non-stripe genomic regions at 5 kb resolution; Middle: mean 3D distance matrices of corresponding regions from scMicro-C; bottom: sorted scMicro-C contact profiles.

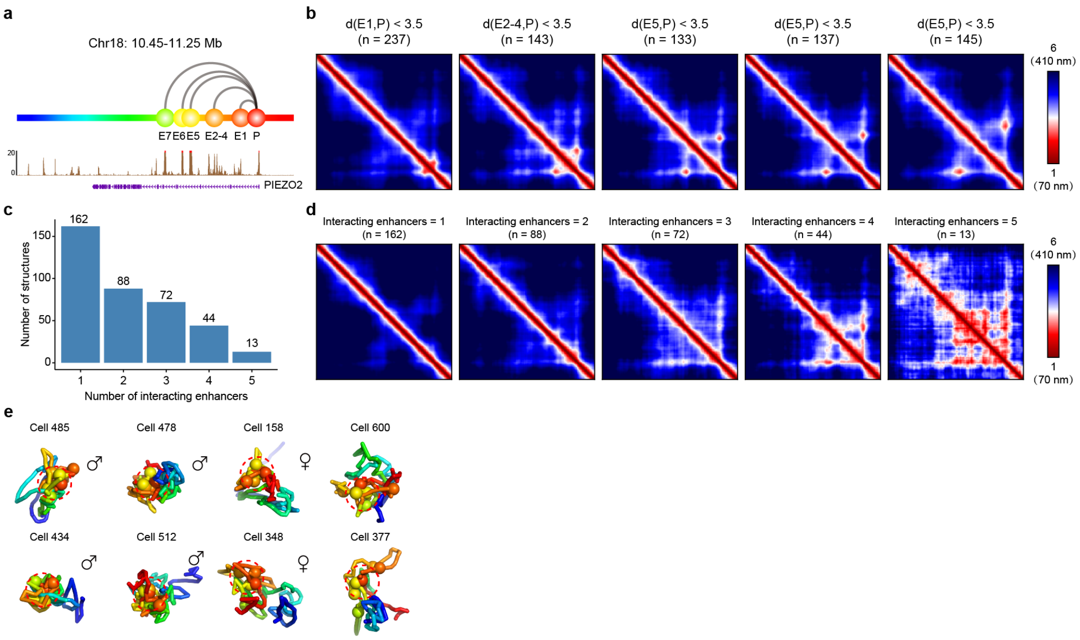

Supplementary Fig. 6. Prevalence and potential mechanism of multi-enhancer hubs. a, Schematics illustrating the genomic loci selected for single-cell 3D genome analysis at PIEZO2 gene locus. **b**, Mean 3D distance matrices of single-cell 3D structures forming corresponding E–P loops. **c**, Histogram summarizing single-cell 3D genome structures forming multi-enhancer hubs with indicated number of enhancers. **d**, Mean 3D distance matrices of single-cell 3D genome structures forming multi-enhancer hubs with indicated number of enhancers. **e,** Representative structures forming multi-enhancer hub at PIEZO2 gene region, the hubs are highlighted.

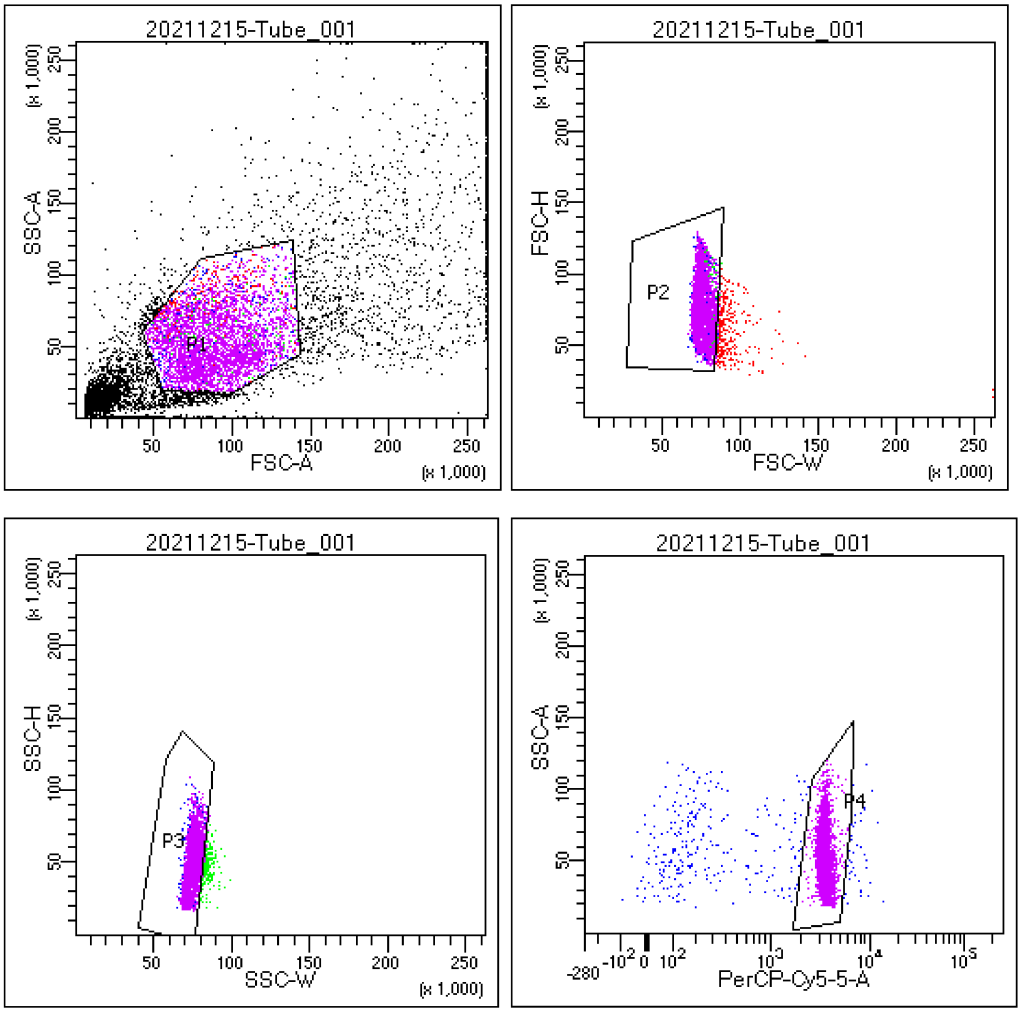

Supplementary Fig. 7. FACS sorting single-cell sequential gating strategy. Cells were first isolated from debris based on FSC-A and SSC-A, then cell clamps were removed by sequential FSC-W and SSC-W, finally single-nuclei were selected based on 7-AAD positive staining.

Supplementary protocol

Buffer preparation

1. Wash buffer (store at 4 °C)

Reagents	Volume	Final conc.
10% BSA (MACS 130-091-376)	50 µL	10 mg/mL
1x PBS	50 mL	

2. Micro-C buffer 1 (store at -20 °C)

Reagents	Volume (µL)	Final conc.
5 M NaCl (Invitrogen AM9760G)	50	50 mM
1 M Tris pH 7.5 (Invitrogen 15567027)	50	10 mM
1 M MgCl2 (Invitrogen AM9530G)	25	5 mM
1 M CaCl2 (Sigma 21115)	5	1 mM
5% Digitonin (Sigma D141-100MG)	50	0.05%
100x protease inhibitor cocktail (Roche 4693159001)	50	1x
Nuclease-free H2O	4770	

3. Micro-C buffer 2 (store at -20 °C)

Reagents	Volume (µL)	Final conc.
5 M NaCl (Invitrogen AM9760G)	100	50 mM
1 M Tris pH 7.5 (Invitrogen 15567027)	100	10 mM
1 M MgCl2 (Invitrogen AM9530G)	100	10 mM
Nuclease-free H2O	9700	

4. Micro-C Buffer 3 (store at -20 °C)

Reagents	Volume (µL)	Final conc.
1 M Tris pH 7.5 (Invitrogen 15567027)	500	50 mM
1 M MgCl2 (Invitrogen AM9530G)	100	10 mM
Nuclease-free H2O	9400	

5. 2x Triton lysis buffer (store at -20 °C)

Reagents	Volume (µL)	Final conc.
1 M Tris pH 8.0 (Invitrogen AM9855G)	100	20 mM
5 M NaCl	40	40 mM
0.5 M EDTA (Invitrogen AM9260G)	20	2 mM

10% Triton X-100 (Sigma 93443)	100	0.2%
1 M DTT (Sigma 646563)	150	30 mM
Nuclease-free H2O	4590	

6. 2x Trans8k buffer (store at -20 °C)

Reagents	Volume (µL)	Final conc.
1 M TAPS pH 8.5 (Boston BioProducts, BB-2375)	100	20 mM
1 M MgCl2	50	10 mM
40% PEG 8000 (Sigma, P1458)	2000	16%
Nuclease-free H2O	2850	

Part I Dual Crosslinking

1. Freshly prepare 1% Paraformaldehyde by add 1 mL 32% PFA (EMS 15714, final 1%) to 31 mL 1x PBS.
2. Freshly Prepare 3 mM DSG (Thermo Fisher 20593).
   a) Prepare 300 mM DSG by dissolving 50 mg DSG in 511 µL DMSO,
   b) Add 300 µL 300 mM DSG to 29.7 mL 1 x PBS.
3. Harvest cells.
   a) Collect cell growth with appropriate density and high viability.
   b) Wash cells once with 1x PBS.
4. Resuspend cell pellets in 1% PFA to a final concentration of 1 million cell per mL, and incubate at room temperature with rotation for 10 min.
5. At the end of incubation, add 1 M Tris-HCl pH 7.5 (Invitrogen, 15567027) to a final concentration of 0.1 M, and incubate at RT for 5 min.
6. Centrifuge at 1000 x g for 5 min at 4°C in a swing bucket centrifuge.
7. Remove the supernatant, then wash twice with 5 mL Wash buffer.
8. Then resuspend in 3 mM DSG to a final concentration of 1 million cell per mL, and incubate at room temperature with rotation for 45 min.
9. At the end of incubation, add Tris-HCl pH 7.5 to a final concentration of 0.1 M, and incubate at RT for 5 min.
10. Centrifuge at 1000 x g for 5 min at 4°C in a swing bucket centrifuge.
11. Wash twice with 5 mL Wash buffer.
12. Aliquot 1 million cell per tube, and store at -80°C.

Part II Modified Micro-C

1. Dilute MNase.
   a) Make MNase Dilution Buffer (10 mM Tris-HCl pH 7.5, 50 mM NaCl, 1 mM EDTA, 50% Glycerol).
   b) Aliquot 10,000 Unit MNase (NEB M0247S, 2,000 U/µL) to 500 µL MNase DilutionBuffer to a final concentration of 20 U/µL.
   c) Aliquot and store at -80°C.
2. Thaw 5 tubes of cell pellets on ice.

3. Resuspend in 100 μL Micro-C Buffer 1, incubate on ice for 20 min, and pipette every 5 min.
4. Centrifuge at 800 x g at 4°C for 5 min. Carefully discard supernatant.
5. MNase titration.
   a) Resuspend the pellet in 100 μL Micro-C Buffer 1.
   b) Add corresponding amount of MNase to each tube, if start with 1 million cells,we will test 50U, 100U, 150U, 200U, 400U, (a titration, typically for 1 million cells, the optimal MNase is round 100-200U).
   c) Mix thoroughly, then incubate at 37°C for 10 min on a thermomixer.
6. At the end of incubation, add 2 μL 0.5 M EGTA to stop the reaction.
7. Take 5 μL to check digestion efficiency, remaining centrifuge at 800 x g at 4°C for 5 min.
8. Carefully discard the supernatant, and wash once with 500 μL Micro-C Buffer 2.
9. Store at -80°C (stable for up to several weeks) or proceed to End Repair.
10. Check Digestion Efficiency
    a) Add 35 μL 1 x PBS to 5 μL digested nuclei, 5 μL 10% SDS (Sigma, 71736-100ML), and 5 μL 20 mg/mL ProteaseK(QIAGEN, 19131).
    b) Incubate at 65°C for 2 hr.
    c) Purify with DNA clean column (ZYMO, D4014).
    d) Run capillary electrophoresis (Fragment Analyzer/TepeStation/Bioanalyzer) to analyze digestion efficiency.
    e) Determine the optimal digestion efficiency, with more than 60% of fragments belongs mono- and di-nucleosome.
11. Thaw cell pellet on ice.
12. Prepare 0.3% SDS by adding 15 μL 10% SDS to 475 μL Micro-C Buffer 2 to a final concentration of 0.3%.
13. Resuspend the cell pellets in 50 μL 0.3% SDS.
14. Incubate at 65°C for 10 min on a thermomixer with 800 rpm shaking.
15. At the end of incubation, add 50 μL 3% Triton X-100 to quench SDS. Incubate at 37°C for 15 min.
16. Centrifuge at 800 x g for 5 min, carefully remove supernatant.
17. Prepare End Repair Mix 1 (45 μL each sample).

Reagents	Per Rxn	Final conc.
10 X NEBuffer 2.1 (NEB B7202S)	5	1x
100 mM ATP (Thermo R0441)	1	2 mM
100 mM DTT	2.5	5 mM
10 U/μL T4 PNK (NEB M0201S)	2.5	0.5 U/μL
Nuclease-free H2O	34	

18. Resuspend in 45 μL End Repair Mix1, and incubate at 37°C for 15 min on a thermomixer with 800 rpm shaking.
19. At the end of incubation, add 5 μL 5 U/μL Klenow Fragment (NEB M0210S),incubate at 37°C for another 15 min.
20. Prepare End Repair Mix2 (25 μL each sample)

Reagents	Per Rxn	Final conc.

10 X T4 DNA Ligase Buffer (NEB B0202S)	2.5	1x
10 mM (each) dNTP (NEB N0447S)	0.5	0.67 mM
Recombinant Albumin (NEB, B9200S)	0.125	
Nuclease-free H2O	21.875	

21. Add 25 µL End Repair Mix2 to each tube, incubate at 23°C for 45 min on a thermomixer (shake 30 s and pause 2 min).
22. Prepare Ligation Buffer (250 µL per sample):
    a) 135 µL water
    b) 25 µL 10 X T4 DNA Ligase Buffer (NEB B0202S, or comes with NEB M0202S)
    c) 2.5 µL 20 mg/mL BSA (NEB B9000S) (final: 0.1 mg/mL)
    d) 12.5 µL 400 U/µL T4 DNA Ligase (NEB M0202S) (final: 20 U/µL, or 5000 U)
    e) Pipette to mix.
23. Add 175 µL Ligation mix to end repaired nuclei.
24. Rotate at RT for 4 hr, followed by 16°C overnight.
25. Check Ligation Efficiency.
    a) Take 25 µL ligation product above sample to check ligation efficiency.
    b) Add 65 µL 1 x PBS, 5 µL 10% SDS (Sigma, 71736-100ML), and 5 ul 20 mg/mL ProteaseK(QIAGEN, 19131).
    c) Incubate at 65°C for 2 hr.
    d) Purify with DNA clean column (ZYMO, D4014).
    e) Run capillary electrophoresis (Fragment Analyzer/TepeStation/Bioanalyzer) to analyzeligation efficiency.

Part III single-cell amplification.

1. Primers.
    a) META20 sequence

Primer	Sequence
META20-1	AGAAGCCGTGTGCCGGTCTAAGATGTGTATAAG
META20-2	ATCGTGCGGACGAGACAGCAAGATGTGTATAAG
META20-3	AATCCTAGCACCGGTTCGCCAGATGTGTATAAG
META20-4	ACGTGTTGCAGGTGCACTCGAGATGTGTATAAG
META20-5	ACACCACACGGCCTAGAGTCAGATGTGTATAAG
META20-6	TGGACAATCACGCGACCAGCAGATGTGTATAAG
META20-7	TCATCTAACGCGCACCGTGCAGATGTGTATAAG
META20-8	TTCGTCGGCTCTCTCGAACCAGATGTGTATAAG
META20-9	TGGTGGAGCGTGCAGACTCTAGATGTGTATAAG
META20-10	TATCTTCCTGCGCAGCGGACAGATGTGTATAAG
META20-11	CTGACGTGTGAGGCGCTAGAAGATGTGTATAAG
META20-12	CCATCATCCAACCGGCTTCGAGATGTGTATAAG
META20-13	CACGAGAAGCCGTCCGCTTAAGATGTGTATAAG
META20-14	CGTACGTGCAACACTCCGCTAGATGTGTATAAG
META20-15	CTTGGTCAGGCGAGAAGCACAGATGTGTATAAG

META20-16	GGCGTGATCAGTGCGTGGATAGATGTGTATAAG
META20-17	GAGCGTTTGGTGACCGCCATAGATGTGTATAAG
META20-18	GCCTGCGGTCCATTGACCTAAGATGTGTATAAG
META20-19	GTAAGCCACTCCAGCGTCACAGATGTGTATAAG
META20-20	GATCTGTTGCGCGTCTGGTGAGATGTGTATAAG

b) META Transposon

Primer	Sequence
META Transposon	5'-[META sequence]-AGATGTGTATAAGAGACAG-3'
ME	5'-/phos/-CTGTCTCTTATACACATCT-3'

c) Preamp primer

Primer	Sequence
META_preamp	5'-[META sequence]-AGATGTGTATAAGAGACAG-3'

d) META40 primer

Primer	Sequence
META20_Read1	ACACTCTTTCCCTACACGACGCTCTTCCGATCT-[META20]-AGATGTGTATAAG
META20_Read2	GACTGGAGTTCAGACGTGTGCTCTTCCGATCT-[META20]-AGATGTGTATAAG

e) ssCarrier DNA.

Primer	Sequence
Carrier ssDNA	TCAGGTTTTCCTGAA

2. Cell lysis.
   a) Dilute 2x Triton lysis to 1x and supplemented with 1.5 mg/mL QIAGEN Protease and 0.5 μM Carrier ssDNA DNA.
   b) Aliquot 2 μL to 96-well plates using multi-channel pipette.
   c) FACS sort single cell to each well.
   d) Incubate at 50°C, 1 hr; 65°C, 1hr and 70°C, 15 min.
   e) Stored at -80C° or proceed to transposon-based amplification.
3. Prepare META transposome.
   a) Dissolve META20 transposon and ME to 100 μM.
   b) Mix 20 META transposons with equal volume.
   c) Mix META transposons with ME, and perform annealing.
   d) Mix equal molar of annealed META transposons with Tn5.
   e) Incubate at 23°C for 30 min.
   f) Dilute to 1.25 μM META20 transposome.
   g) Further dilute to 0.125 μM, store at -80°C.
4. Tagmentation.
   a) Prepare transposition mix

Reagents	Per Rxn	110 Rxn	Final conc.
2x Trans8k	4	550	1x
Diluted META20 transposome (30x-50x)	1	110	~0.3 nM

Nuclease-free H2O	1	220	

b) Aliquot 6 µL to each well via multi-channel pipette.
c) Seal the plate, mix thoroughly by vortex.
d) Incubate at 55°C for 10 min.

5. Tn5 Stop.
a) Prepare Tn5 Removal Mix (250 mM NaCl, 37.5 mM EDTA, 2 mg/mL QIAGEN protease).
b) Add 2 µL Tn5 Removal Mix to each well, seal the plate and mix thoroughly by vortex.
c) Incubate as:
50C°, 30 min
70C°, 15 min

6. Pre-amplification.
a) Prepare Preamplification Mix (15 µL each well, below recipe for 1 96-well plate)

Reagents	Per Rxn	110 Rxn	Final conc.
2 X Q5 High-fidelity Master Mix (NEB M0492L)	12.5	1375	1x
100 µM META_preamp	0.5	55	0.1 µM each
100 mM MgCl2	0.5	55	
Nuclease-free H2O	1.5	165	

b) Aliquot 15 µL to each well by milt-channel pipette.
c) Seal the plate and mix thoroughly by vortex.
d) Incubate as
72C, 5min
98C, 30s
11 cycles [98C, 10s, 65C, 1min, 72C, 2min]
72C, 5min

7. Add 1 µL EXOI to each well and incubate as
37°C, 30 min;
80°C, 20 min
8. Dissolve META20_Read1 and META20_Read2 to 50 µM, then mix with equal volume, label as META40 primer.
9. Adaptor incorporation.
a) Prepare adaptor incorporation mix

Reagents	Per Rxn	110 Rxn	Final conc.
2 X Q5 High-fidelity Master Mix (NEB M0492L)	3	330	1x
50 µM META40	2	220	0.1 µM each

b) Aliquot 5 µL to each well, seal the plate, mix thoroughly by vortex.
c) Incubate as
98°C, 30 s;
2 cycles [98°C, 10 s; 65°C, 1 min; 72°C, 2 min]
72C, 5 min

10. Add 1 µL EXOI to each well and incubate as

37°C, 30 min;
80°C, 20 min

11. Library preparation.
    a) Add 2 µL i5 index primer and 2 µL i7 index (Vazyme, N321/322) primer to each well to make unique sample index combination.
    b) Add 5 µL 2 X Q5 High-fidelity Master Mix to each well.
    c) Seal the plate, mix thoroughly by vortex.
    d) Incubate as
       98°C, 30 s;
       2 cycles [98°C, 10 s; 65°C, 1 min; 72°C, 2 min]
       72C, 5 min
12. Pool whole plate, purify with ZYMO DCC5, elute with 50 µL elution buffer.
13. Further size selection with 0.7x SPRI beads, elute with 40 µL elution buffer.
14. Quantify the library via qPCR.
15. Sequence on Illumina NovaSeq 6000/Xplus platform with paired-end, 6 million reads/cell.
