# Supplementary material for: Single-cell Micro-C profiles 3D genome structures at high resolution and characterizes multi-enhancer hubs
Source: Nat Genet. 2025 Jul 2;57(7):1777–86. doi: 10.1038/s41588-025-02247-6 (PMC12283367; doi:10.1038/s41588-025-02247-6)
Supplement: Supplementary file 2 — Reporting Summary [file 41588_2025_2247_MOESM2_ESM.pdf]

nature portfolio

Corresponding author(s): Xiaoliang Sunney Xie

Last updated by author(s): Mar 18, 2025

Reporting Summary

Nature Portfolio wishes to improve the reproducibility of the work that we publish. This form provides structure for consistency and transparency in reporting. For further information on Nature Portfolio policies, see our Editorial Policies and the Editorial Policy Checklist.

Statistics

For all statistical analyses, confirm that the following items are present in the figure legend, table legend, main text, or Methods section.

n/a	Confirmed	
☐	☒	The exact sample size (*n*) for each experimental group/condition, given as a discrete number and unit of measurement
☐	☒	A statement on whether measurements were taken from distinct samples or whether the same sample was measured repeatedly
☐	☒	The statistical test(s) used AND whether they are one- or two-sided<br>*Only common tests should be described solely by name; describe more complex techniques in the Methods section.*
☒	☐	A description of all covariates tested
☒	☐	A description of any assumptions or corrections, such as tests of normality and adjustment for multiple comparisons
☐	☒	A full description of the statistical parameters including central tendency (e.g. means) or other basic estimates (e.g. regression coefficient) AND variation (e.g. standard deviation) or associated estimates of uncertainty (e.g. confidence intervals)
☐	☒	For null hypothesis testing, the test statistic (e.g. *F*, *t*, *r*) with confidence intervals, effect sizes, degrees of freedom and *P* value noted<br>*Give P values as exact values whenever suitable.*
☒	☐	For Bayesian analysis, information on the choice of priors and Markov chain Monte Carlo settings
☒	☐	For hierarchical and complex designs, identification of the appropriate level for tests and full reporting of outcomes
☒	☐	Estimates of effect sizes (e.g. Cohen's *d*, Pearson's *r*), indicating how they were calculated

Our web collection on statistics for biologists contains articles on many of the points above.

Software and code

Policy information about availability of computer code

Data collection	FACSDiva.
Data analysis	The code used in this study is publicly available at GitHub (https://github.com/tanlongzhi/dip-c and https://github.com/lh3/ hickit). BWA-MEM (v0.7.17), cooler (v.0.8.11), Juicer (v1.19.02), cooltools (v0.5.2), deepTools (v3.5.2), chromosight (v1.6.2), coolpuppy (v1.0.0), Stripenn (v1.1.65.7), StripeCaller (v0.1.0), DANPOS2 (v2.2.2)

For manuscripts utilizing custom algorithms or software that are central to the research but not yet described in published literature, software must be made available to editors and reviewers. We strongly encourage code deposition in a community repository (e.g. GitHub). See the Nature Portfolio guidelines for submitting code & software for further information.

Data

Policy information about availability of data

All manuscripts must include a data availability statement. This statement should provide the following information, where applicable:

- Accession codes, unique identifiers, or web links for publicly available datasets
- A description of any restrictions on data availability
- For clinical datasets or third party data, please ensure that the statement adheres to our policy

Raw next generation sequencing data were deposited on Sequence Read Archive (SRA; https://www.ncbi.nlm.nih.gov/sra) with accession number PRJNA78. All processed data were deposited on the Gene Expression Omnibus (GEO; http://www.ncbi.nlm.nih.gov/

geo/) with accession number GSE281150 (Superseries). The subseries includes GSE225201 for bulk Micro-C, GSE278191 for scMicro-C 1000U, GSE279479 for scMicro-C 600U, GSE279583 for scMicro-C 800U, GSE279732 for scMicro-C 200U, GSE280482 for SDS test, GSE192759 for scMicro-C pool. Data were mapped to genome assembly GRCh38.

Research involving human participants, their data, or biological material

Policy information about studies with human participants or human data. See also policy information about sex, gender (identity/presentation), and sexual orientation and race, ethnicity and racism.

Reporting on sex and gender	Not applicable. There is no human participants involved in this study.
Reporting on race, ethnicity, or other socially relevant groupings	Not applicable.
Population characteristics	Not applicable.
Recruitment	Not applicable.
Ethics oversight	Not applicable.

Note that full information on the approval of the study protocol must also be provided in the manuscript.

Field-specific reporting

Please select the one below that is the best fit for your research. If you are not sure, read the appropriate sections before making your selection.

☒ Life sciences ☐ Behavioural & social sciences ☐ Ecological, evolutionary & environmental sciences

For a reference copy of the document with all sections, see nature.com/documents/nr-reporting-summary-flat.pdf

Life sciences study design

All studies must disclose on these points even when the disclosure is negative.

Sample size	No statistical method was used to predetermine sample size. For single-cell Micro-C data, there were 810 cells sequenced. For bulk Micro-C, there were at least two replicates were sequenced for each condition. No sample size estimation is needed. We
Data exclusions	52 scMicro-C cells were excluded due to low quality.
Replication	We have extensively demonstrated the robustness and reproducibility of our methodology. There were at least two independent biological replicates were performed for all bulk sequencing samples (bulk Micro-C). For single-cell data, five batches generated. All replicates were successfully amplified and sequenced.
Randomization	Not applicable. There is no treatment/perturbation groups in this study.
Blinding	Not applicable. Blinding was not required since our sample is taken from normal cultured cell line, there is no treatment or perturbation.

Reporting for specific materials, systems and methods

We require information from authors about some types of materials, experimental systems and methods used in many studies. Here, indicate whether each material, system or method listed is relevant to your study. If you are not sure if a list item applies to your research, read the appropriate section before selecting a response.

Materials & experimental systems

n/a	Involved in the study	
☒	☐	Antibodies
☐	☒	Eukaryotic cell lines
☒	☐	Palaeontology and archaeology
☒	☐	Animals and other organisms
☒	☐	Clinical data
☒	☐	Dual use research of concern
☒	☐	Plants

Methods

n/a	Involved in the study	
☒	☐	ChIP-seq
☐	☒	Flow cytometry
☒	☐	MRI-based neuroimaging

Eukaryotic cell lines

Policy information about cell lines and Sex and Gender in Research

Cell line source(s)	GM12878 (Coriell Institute) is a EBV-transformed B lymphocyte from a female.
Authentication	All cell lines were validated with morphology and gene expression and other epigenetic states with published datasets.
Mycoplasma contamination	Mycoplasma contamination test is negative.
Commonly misidentified lines (See ICLAC register)	No commonly misidentified cell lines were used in the study.

Plants

Seed stocks	No plant material was used in this study.
Novel plant genotypes	Not applicable.
Authentication	Not applicable.

Flow Cytometry

Plots

Confirm that:

☒ The axis labels state the marker and fluorochrome used (e.g. CD4-FITC).

☒ The axis scales are clearly visible. Include numbers along axes only for bottom left plot of group (a 'group' is an analysis of identical markers).

☒ All plots are contour plots with outliers or pseudocolor plots.

☒ A numerical value for number of cells or percentage (with statistics) is provided.

Methodology

Sample preparation	GM12878 cells followed Micro-C procedure were filtered with 30 um cell strainer, then stained with 7-AAD to distinguish nuclei from debris.
Instrument	BD, FACS Aria SORP
Software	BD FACSDiva v9.0 Software
Cell population abundance	All nuclei were sorted without biased, the 7-AAD-negative nuclei was selected.
Gating strategy	Nuclei were distinguished from debris based on FSC-A and SSC-A, then the multiplets were removed by two step gating of FSC-W and FSC-H, SSC-W and SSC-H. Then nuclei were selected based on PerCP-cy5-5-A.

☒ Tick this box to confirm that a figure exemplifying the gating strategy is provided in the Supplementary Information.
